# Evaluating disparities in code status designation among patients admitted with COVID-19 at a quaternary care center early in the pandemic

**DOI:** 10.1097/MD.0000000000034447

**Published:** 2023-07-28

**Authors:** Peter K. Olds, Nicholas Musinguzi, Benjamin P. Geisler, Pankaj Sarin, Jessica E. Haberer

**Affiliations:** a Massachusetts General Hospital/Harvard Medical School, Boston, MA; b Global Health Collaborative, Mbarara University of Science and Technology, Mbarara, Uganda; c Ludwig Maximilian University Munich, Munich, Germany; d Brigham and Women’s Hospital/Harvard Medical School, Boston, MA.

**Keywords:** code status, COVID-19, disparities, goals of care, inpatient

## Abstract

The COVID-19 pandemic has highlighted disparities in outcomes by social determinants to health. It is unclear how much end-of-life discussions and a patient’s decision about code status (“do not resuscitate,” do not resuscitate, or “comfort measures only,” [CMO] orders) might contribute to in hospital disparities in care, especially given know racial inequities in end-of-life care. Here, we looked at factors associated with code status orders at the end of hospitalization for patients with COVID-19. We conducted a retrospective chart review of all patients who presented to the Emergency Department of a large quaternary hospital between 8 March and 3 June 2020. We used logistic regression modeling to quantify the degree to which social determinants of health, including race, ethnicity, area deprivation index (ADI), English as a primary language, homelessness, and illicit substance use might impact the likelihood of a particular code status at the end-of a patient’s hospitalization, while controlling for disease severity. Among social determinants to health, only white race (odds ratio [OR] 2.0; *P* = .03) and higher ADI (OR 1.2; *P* = .03) were associated with having a do not resuscitate or a CMO order. Additionally, we found that patients with white race (OR 2.9; *P* = .02) were more likely to carry a CMO order. Patient race and ADI were associated with different code status orders at the end of hospitalization. Differences in code status might have contributed to disparities in COVID-19 outcomes early in the pandemic, though further investigations are warranted.

## 1. Introduction

The COVID-19 pandemic has revealed significant disparities in medical care in the U.S. along the lines of race, ethnicity, and other social determinants of health including socioeconomic status, housing status, primary language, substance use, and severe psychiatric illness.^[1–3]^ Significant differences documented thus far have included risk of hospitalization, intubation and mechanical ventilation, intensive care unit admission, and mortality.^[4,5]^ Better understanding of these disparities is critical towards addressing future COVID-19 waves as well as improving equity of care more broadly.

Given the high mortality rates early in the COVID-19 pandemic, differing end-of-life discussions and associated decisions about desired levels of care, such as code status, may have contributed to differences in outcomes. End-of-life care is especially important as it is well-documented that there are significant racial disparities in the US around end-of-life care. Racial minorities are less likely to receiving care consistent with preferences or have their pain adequately addressed, and suffer from underutilization of palliative and end-of-life services.^[6]^ Additionally, there are well-documented disparities in end-of-life care by socioeconomic status, with poor patients less likely to have access to palliative care, hospice, and quality nursing home care.^[7]^

Code status refers to whether a patient would like to receive cardiopulmonary resuscitation and/or endotracheal intubation in case of an appropriate clinical indication—most often in a medical emergency. Patients who have elected “do not resuscitate” (DNR) and/or “do not intubate” (DNI) orders, do so often after discussions with healthcare providers when such interventions are felt to provide more harm than benefit. “Comfort measures only” (CMO) is an extension of DNR/DNI status, where the goal of medical care is focused purely on patient comfort and symptom management, rather than clinical improvement or cure. Given that transfer to the intensive care unit (ICU) and intubation are important interventions for severe COVID-19 infection, differences in patient code status may contribute to differences in COVID-19 outcomes.

Previous work looking at code status changes for COVID-19 patients has found mixed results. Some authors report that code status orders were unchanged during the pandemic when compared to before the pandemic,^[8]^ while others showed increased DNR orders, earlier end-of-life discussions, and a higher demand for palliative care services.^[9,10]^ In looking at social determinants, Epler et al^[12]^ found that having Medicaid as insurance was a predictor for having a DNR order, and Barnato et al^[11]^ found that Black and Hispanic patients were less likely to have DNR orders. Additionally, ICU patients with a preferred language other than English have been found to be less likely to have a DNR order.^[13]^ However, no study to date has looked at a broader range of social determinants on all admitted patients and how they might impact COVID-19 patients code status.

Our objective was therefore to analyze predictors of hospital code status at the end-of hospital stay for patients admitted with COVID-19 infection. Specifically, we examined multiple social determinants of health, including race, ethnicity, socioeconomic status, illicit drug use, primary language, and housing status.

## 2. Methods

### 2.1. Study design

We conducted a retrospective analysis using the Massachusetts General Hospital (MGH) COVID-19 Data Registry, which includes confirmed SARS-CoV-2-infected patients who presented to the MGH emergency department (ED). The database was compiled with both data extraction from the electronic medical record as well as manual chart reviews. Trained reviewers collected demographics, comorbid conditions, medications, and epidemiological risk factors for SARS-CoV-2 infection. Patient data was collected from each day of their hospital stay and each patient had 28 days of follow-up from the date of presentation to evaluate for mortality.

Additionally, the area deprivation index (ADI) score was downloaded on July 28, 2020 from the Health Services Advisory Group website as a measure of social determinants of health.^[14]^ The ADI is composed of 17 measures covering education, employment, housing-quality, and poverty and drawn from both the National Census and American Community Survey data and provides a disparity score by 9-digit zip code.^[15,16]^ The ADI is scored out of 10 and is inversely related to socioeconomic status (i.e., 10 indicating the lowest socioeconomic status). Because our database only included patients 5-digit zip codes, we averaged ADI scores within each 5-digit zip code to provide a score for each patient.

### 2.2. Setting and participants

This study took place at MGH in Boston, Massachusetts, a 999-bed quaternary referral teaching hospital and a major referral center for COVID-related care throughout the pandemic. At MGH, a code status order is required for hospital admission and is therefore addressed on every admission. We included all patients 18 years and older who presented to the MGH ED between 8 March and 3 June 2020 who; Had SARS-CoV-2 infection confirmed via polymerase-chain reaction nasopharyngeal swab testing and; Had documented code status data.

### 2.3. Analysis

Participant characteristics were summarized descriptively. Patients had code status orders of Full Code, DNR, DNI, DNR/DNI, or CMO. All patients with a DNR, DNI, or DNR/DNI were combined into 1 category given the similarities in these code statuses and very low numbers of patients with either DNR or DNI code status. A patient’s final code status was the code status order at the end-of hospital stay, and did not always reflect a change in code status order from admission. Comparisons between patients whose last recorded code status during the current hospitalization was Full Code (i.e., no restrictions on care provision aimed at cure), DNR/DNI, or CMO were made with Dunn test and Pearson chi-square tests for continuous and categorical variables, respectively. All tests were 2-sided and a *P* value <.05 was considered statistically significant (see Table S2 and S2, Supplemental Digital Content, http://links.lww.com/MD/J343, which shows the results of univariate analysis).

A multivariable logistic regression was fitted for primary outcomes of full code versus other code status and CMO versus other code status. Key associations of interest were race, ethnicity, ADI, English as a primary language, homelessness, and illicit substance use (i.e., opiates, cocaine, methamphetamine). We also evaluated for age, comorbidities (i.e., history of lung, renal, lung disease, stroke, heart failure, cancer, and Human immunodeficiency virus), body mass index, ICU admission, need for mechanical ventilation, and clinical severity. We evaluated disease severity using clinical severity scores (sequential organ failure assessment [SOFA], Charlson Comorbidity Index) and laboratory markers found in other risk severity scores,^[17,18]^ specifically, C-reactive protein (mg/L), ferritin (ug/L), D-dimer (ng/mL), creatine kinase (U/L), troponin (ng/L), procalcitonin (ng/mL), absolute lymphocyte count (K/mL), blood urea nitrogen (mg/dL). To build our regression models, we first included a priori variables based on clinical understanding (i.e., age, sex, SOFA, C-reactive protein, ferritin, troponin, ICU admission, and mechanical ventilation), and then added variables that were significant on univariable analysis. We also controlled for ICU admission, mechanical ventilation, and clinical severity scores (i.e., SOFA^[19]^ and Charlson Comorbidity Index ^[20]^); these latter variables were excluded if they showed significant co-linearity (variance inflation factors over 10). We used stepwise, backward selection for our logistic regression model, using a *P* value of .2 as a cutoff to remove variables. Potential interaction between significant variables was explored.

Additionally, as a third outcome, we evaluated on what hospital day a patient’s code status was changed for the last time during an admission. We made a histogram plot of this data and calculated the mean day of final code status change. Patients with missing data were excluded from analysis. All data were analyzed using Stata IC 16.0 (StataCorp LLC, College Station, TX).

### 2.4. Ethical approval

Study approval was obtained from the Mass General Brigham Healthcare Institutional Review Board (2020P001789).

## 3. Results

Of the 1302 total visits recorded of patients with COVID-19 who presented to the ED during this period, 1163 had a code status documented. As shown in Table 1, their mean age was 60 years (standard deviation 18), and 42.8% were women. Medical comorbidities were common, 51.5% with hypertension, 34.0% with diabetes, 30.0% with lung disease, 16.6% with chronic kidney disease, and 1.4% with Human immunodeficiency virus. White patients made up 38.3% of the population, with Hispanic and African American patients comprising the second and third largest populations at 36.4% and 10.7%, respectively. Primary language was recorded as English for 41.4% and a language other than English for 30.6% of the population. Missing data was common with 8.6% of participants missing race data and 28.0% missing language data. The average ADI was 6.0 (standard deviation 5.9–6.1). Patients documented as homeless made up 3.1% of the population; 4.1% reported using illicit substances (i.e., opiates, methamphetamine, cocaine). At the time of hospital admission, most patients had full code status (90.2%), with patients initially being DNR/DNI and CMO comprising 8.9% and 0.9%, respectively (Table 1). At the end-of hospitalization, patients with a full code, DNR/DNI, and CMO orders represented 73.3%, 14.7%, and 12.0% respectively (Table 2).

**Table 1:** Descriptive statistics of the patient population at hospital admission. This table shows the descriptive statistics for patients based on their code status at time of hospital admission.

	Overall	Code status at time of hospital admission			
		Full Code	DNR/DNI	CMO	*P* value
Total (N, %)	1163	1049 (90.2)	104 (8.9)	10 (0.9)	
Age, mean, yrs (SD)	60 (18)	58 (17)	80 (10)	91 (7)	<.001
Female sex (N, %)	498 (42.8)	440 (41.9)	52 (50.0)	6 (60.0)	.16
Race/ethnicity (N, %)					
White	445 (38.3)	349 (33.3)	88 (84.6)	8 (80.0)	<.001
Black	125 (10.7)	122 (11.6)	3 (2.9)	0 (0.0)	.01
Asian	43 (3.7)	40 (3.8)	2 (1.9)	1 (10.0)	.35
Hispanic	423 (36.4)	415 (39.6)	7 (6.7)	1 (10.0)	<.001
Other	27 (2.3)	25 (2.4)	2 (1.9)	0 (0.0)	.85
Missing	100 (8.6)	98 (9.3)	2 (1.9)	0 (0.0)	.02
Comorbidities (N, %)					
Hypertension	599 (51.5)	511 (48.7)	79 (76.0)	9 (90.0)	<.001
Diabetes	395 (34.0)	350 (33.4)	43 (41.4)	2 (20.0)	.17
Lung disease	349 (30.0)	296 (28.7)	48 (46.6)	5 (50.0)	<.001
Kidney disease	193 (16.6)	154 (15.0)	35 (34.7)	4 (40.0)	<.001
HIV infection (N, %)	16 (1.4)	14 (1.3)	2 (1.9)	0 (0.0)	.83
Homeless (N, %)	36 (3.1)	35 (3.3)	1 (1.0)	0 (0.0)	.35
Illicit drug use (N, %)	48 (4.1)	46 (4.4)	2 (1.9)	0 (0.0)	.39
Primary language (N, %)					
English	481 (41.4)	409 (39.0)	66 (63.5)	6 (60.0)	<.001
Non-english	356 (30.6)	341 (32.5)	13 (12.5)	2 (20.0)	<.001
Missing	326 (28.0)	299 (28.5)	25 (24.0)	2 (20.0)	.53
Area deprivation index, mean (95% CI)	6.0 (5.9–6.1)	6.0 (5.9–6.2)	5.8 (5.3–6.2)	5.5 (3.3–7.6)	.41

CI = confidence interval, CMO = comfort measures only, DNR/DNI = do not resuscitate/do not intubate, HIV = human immunodeficiency virus, SD = standard deviation.

**Table 2:** Descriptive statistics of the patient population at end-of hospital stay. This table shows the descriptive statistics for patients based on their code status at time of hospital discharge or death.

	Overall	Code status at end-of hospitalization			
		Full code	DNR/DNI	CMO	*P* value
Total	1163	852 (73.3)	171 (14.7)	140 (12.0)	
Age, mean, yrs (SD)	60 (18)	54 (16)	76 (13)	77 (13)	<.001
Female sex (N, %)	498 (42.8)	392 (46.0)	85 (49.7)	51 (36.4)	.06
Race/ethnicity (N, %)					
White	445 (38.3)	244 (28.6)	112 (65.5)	89 (63.6)	<.001
Black	125 (10.7)	96 (11.3)	17 (9.9)	12 (8.6)	.55
Asian	43 (3.7)	35 (4.1)	3 (1.8)	5 (3.6)	.33
Hispanic	423 (36.4)	378 (44.4)	26 (15.2)	19 (13.6)	<.001
Other	27 (2.3)	22 (2.6)	2 (1.2)	3 (2.1)	.53
Missing	100 (8.6)	77 (9.0)	11 (6.4)	12 (8.6)	.54
Comorbidities (N, %)					
Hypertension	599 (51.5)	370 (43.4)	122 (71.3)	107 (76.4)	<.001
Diabetes	395 (34.0)	273 (32.0)	57 (33.3)	65 (46.4)	.004
Lung disease	349 (30.0)	212 (24.9)	75 (43.9)	62 (44.3)	<.001
Kidney disease	193 (16.6)	96 (11.3)	44 (25.7)	53 (37.9)	<.001
HIV infection, (N, %)	16 (1.4)	12 (1.4)	3 (1.8)	1 (0.7)	.73
Homeless (N, %)	36 (3.1)	33 (3.9)	2 (1.2)	1 (0.7)	.04
Illicit drug use (N, %)	48 (4.1)	41 (4.8)	3 (1.8)	4 (2.9)	.13
Primary language (N, %)					
English	481 (41.4)	305 (35.8)	103 (60.2)	73 (52.1)	<.001
Non-english	356 (30.6)	297 (34.9)	32 (18.7)	27 (10.3)	<.001
Missing	326 (28.0)	250 (29.3)	36 (21.0)	40 (28.6)	.09
Area deprivation index, mean (95% CI)	6.0 (5.9–6.1)	6.1 (5.9–6.2)	5.6 (5.3–6.0)	6.0 (5.6–6.3)	.007

CI = confidence interval, CMO = comfort measures only, DNR/DNI = do not resuscitate/do not intubate, HIV = human immunodeficiency virus, SD = standard deviation.

**Figure 1.**
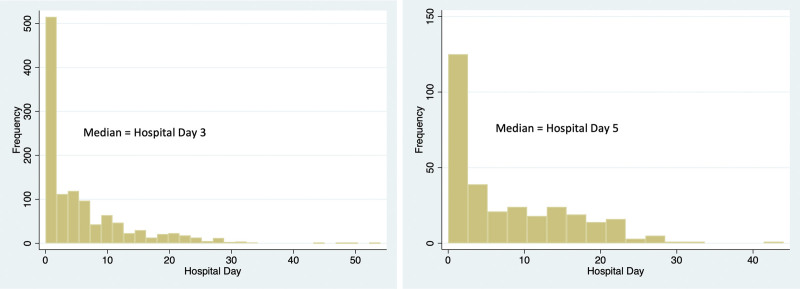
Graphical representation of the timing of final code status change by day of the hospitalization. (A) all patients in the cohort and (B) all patients whose final code status was Do Not Resuscitate/Do Not Intubate (DNR/DNI) or Comfort Measures Only (CMO).

Just over 44% of final code status decisions were made by the first day of a patient’s hospital stay, with the median time to date of change being 3 days into the hospital stay (Fig. 1A). When we focused only on final code status changes of DNR/DNI and CMO, the mean date increased to 5 days, though 1-third of the decisions occurred by hospital day 1 (33.8%) (Fig. 1B).

In univariable analysis, social determinants of health that were associated with having a code status order other than full code were race, ADI, primary language, and homelessness (Table 3). In multivariable analysis, white race [odds ratio (OR) 2.0, confidence interval (CI) 1.1–3.8, *P* = .03] and each increase in unit of the ADI (OR 1.2, CI 1.0–1.3, *P* = .03) were associated with having a DNR or CMO order. No interaction was seen between race and ADI. When looking at social determinants of health associated with a patient being CMO, in univariate analysis we found that race and ADI had significant associations (Table 4). In multivariable analysis, patients with white race (OR 2.9, CI 1.2–6.7, *P* = .02) were more likely to carry a CMO order.

**Table 3:** Variables associated with having a code status other than full code. Logistic regression findings for our key associations for patients who have a code status of either do not resuscitate/do not intubate (DNR/DNI) or comfort measures only (CMO).

	Univariable findings		Multivariable findings*	
	Odds ratio (95% CI)	*P* value	Odds ratio (95% CI)	*P* value
White race (reference: non white)	5.0 (3.8–6.7)	<.001	2.0 (1.1–3.8)	.03
ADI (per unit in the score)	0.9 (0.9–1.0)	.03	1.2 (1.0–1.3)	.03
English primary language (reference: non-english)	2.9 (2.1–4.1)	<.001	1.5 (0.8–3.0)	.24
Homelessness (reference: domiciled)	0.2 (0.1–0.8)	.02	0.6 (0.1–3.3)	.55
Illicit drug use (opiates, cocaine, methamphetamines) (reference: no illicit drug use)	0.5 (0.2–1.0)	.06	0.3 (0.1–1.4)	.13

ADI = area deprivation index, CI = confidence interval.

* The regression model controlled for age, sex, Blood Urea Nitrogen (BUN), D-dimer, ferritin, hypotension, oxygen requirement on hospital admission, endotracheal intubation during hospitalization, history of lung disease, history of diabetes, history of heart failure, and body mass index (BMI) > 30kg/m^2^.

**Table 4:** Criteria associated with having a code status of “comfort measures only.” Logistic regression findings for our key associations for patients with a code status of “comfort measures only”.

	Univariable findings		Multivariable findings*	
	Odds ratio (95% CI)	*P* value	Odds ratio (95% CI)	*P* value
White race (reference: non white)	3.7 (2.5–5.5)	<.001	2.9 (1.2–6.7)	.02
ADI (per unit in the score)	1.0 (0.9–1.1)	.79	1.2 (1.0–1.4)	.07
English primary language (reference: non-english)	2.2 (1.4–3.5)	.001	1.5 (0.6–3.5)	.40
Homelessness (reference: domiciled)	0.2 (0.03–1.5)	.12	2.2 (0.2–20.2)	.47
Illicit drug use (opiates, cocaine, methamphetamines) (reference: no illicit drug use)	0.7 (0.2–1.9)	.42	0.4 (0.1–2.6)	.35

ADI = area deprivation index, CI = confidence interval.

* The regression model controlled for age, sex, C-Reactive Protein (CRP), Blood Urea Nitrogen (BUN), troponin, ferritin, oxygen requirement on admission, Intensive Care Unit (ICU) admission, history of lung disease.

## 4. Discussion

When assessing the effects of social determinants of health and controlling for age, sex, medical comorbidities, ICU admission, and need for mechanical ventilation, we found that white race and a higher ADI (i.e., lower socioeconomic status) were associated with having a final code status of do not resuscitate, do not intubate, or comfort measures only. Additionally, white race was significantly associated with CMO. We also found that a large proportion of decisions occurred within the first days of hospitalization, even when focusing on those patients with a DNR/DNI or CMO order.

The finding that white patients were more likely to have a DNR or CMO code status is in line with the body of literature that highlights racial disparities in code status and end-of-life care in the US. Prior studies have shown that Hispanic and African Americans are less likely to have a DNR or CMO order,^[21,22]^ receive more aggressive therapies in the last month of life,^[23]^ and experience more in hospital deaths and lengths of stay when compared with white patients.^[24]^ Additionally, racial minorities in the US have lower patient and family satisfaction with end-of-life care, are at higher risk for not receiving goal-concordant care, and suffer from underutilization of palliative and end-of-life care services.^[6,25]^ These disparities in end-of-life care are felt to be driven by differences in access to care, lack of culturally adapted end-of-life discussions, and a history of racial injustices in healthcare.^[26,27]^

Several studies evaluating racial disparities in hospital outcomes (i.e., clinical complications, mortality) for COVID-19 patients have shown no difference,^[28–30]^ and a large Veteran Affairs study found that non-Hispanic Black and African American patients had higher rates of hospital complications.^[31]^ It is unclear how much differences in code status might contribute to any possible clinical differences and warrants further investigation.

While we found that a higher ADI, and thus lower socioeconomic status, was associated with a final code status other than full code, the effect was small, and specifically driven by patients with a DNR order and not those with a CMO order. While data is mixed, prior studies have more often shown lower socioeconomic status associated with more aggressive end-of life care rather than less.^[24,32,33]^ Additionally, prior work has found that patients with higher socioeconomic status often have greater access to hospice and thus we would have expected that to be consistent with a higher rate of patients with a CMO order. The differences in code status by socioeconomic status in our study may have been due to differences in patient attitudes given the novel pathogen of SARS-CoV-2. Interestingly, our findings are in line with Epler et al^[12]^ findings that COVID-19 patients on Medicaid – and thus likely lower socioeconomic status – had a higher likelihood of having a DNR order.

End-of-life discussions are complex, often emotionally fraught, and may take a lot of time to occur. Numerous barriers to code status discussions exist in the hospital, including the time required, worries about damaging patient-provider relationship, language differences, and lack of proper frameworks to guide the discussion.^[34,35]^ Additionally, how patients and family approach death and dying are inherently impacted by individual differences and social influences, and culturally adapted discussions have been shown to improve end-of-life decision making.^[36]^

Cultural responsiveness, translation services, and sufficient time and space are critical to ensuring that end-of-life discussions are as effective possible. During the COVID-19 pandemic, surges in hospitalized patients often meant less time, energy, and space available to providers, likely exacerbating disparities in the quantity and quality of end-of-life discussions. Interestingly, studies have shown that early involvement of palliative care-trained providers and more culturally appropriate goals of care discussions minimized racial disparities in end-of-life care.^[37,38]^ Unfortunately, our study did not include data on palliative care consultation or more information on the duration or quality of code status discussions.

A strength of this study is that is among the first to look at disparities in code status decisions during the COVID-19 pandemic. While prior studies have looked primarily at racial disparities, ours focused on several social determinants to health. Specifically, our study used the ADI, which is a powerful tool for evaluating social determinants based on a patient’s zip code. Major limitations of our study are that we have a relatively small cohort from 1 hospital. However, sampling from 1 hospital control for many confounders and intrahospital variations in definitions of DNR and CMO. We also did not have significantly detailed data on psychiatric illness and types of substance use and thus were limited in our analysis of these social determinants of health in this study. Our dataset also lacked data on providers race/ethnicity and languages spoken. Additionally, we did not have data on the number and quality of goals of care discussions or palliative care referrals, both of which have been shown to improve disparities in end-of-life care.^[37,38]^ Finally, our dataset’s timeframe was early in the pandemic, limiting the applicability of our findings. However, given ongoing morbidity from COVID-19 and calls for improved coordination of COVID-19 care, continued research on end-of-life care for COVID-19 patients remains important.^[39–41]^

## 5. Conclusion

Our results indicate that white race and lower socioeconomic status may be associated with a code status other than full code, and white race may be associated with a patient electing a “comfort measures only” code status. Since these disparities in code status may have also contributed to different outcomes, further work is needed to better understand how these differences evolved during the COVID-19 epidemic and to continue a more wide-spread adoption of culturally appropriate end-of-life discussions to help improve these disparities.

## Acknowledgements

The authors would like to thank the time and effort of the dedicated manual chart reviewers and data managers from the MGH COVID registry who made this work possible.

## Author contributions

**Conceptualization:** Peter K. Olds, Benjamin P. Geisler, Jessica E. Haberer.

**Data curation:** Peter K. Olds.

**Formal analysis:** Peter K. Olds.

**Methodology:** Nicholas Musinguzi, Benjamin P. Geisler, Pankaj Sarin.

**Resources:** Pankaj Sarin.

**Supervision:** Jessica E. Haberer.

**Validation:** Nicholas Musinguzi, Jessica E. Haberer.

**Writing – original draft:** Peter K. Olds.

**Writing – review & editing:** Benjamin P. Geisler, Jessica E. Haberer.

## Supplementary Material



## References

[1] Abedi V Olulana O Avula V. Racial, economic, and health inequality and COVID-19 infection in the United States. J Racial Ethn Heal Disparities. 2021;8:732–42. 10.1007/s40615-020-00833-4 PMC7462354 32875535

[2] Harlem G. Descriptive analysis of social determinant factors in urban communities affected by COVID-19. J Public Health (Oxf). 2020;42:466–9. 32530033 10.1093/pubmed/fdaa078 PMC7313894

[3] Hâncean MG Lerner J Perc M. Occupations and their impact on the spreading of COVID-19 in urban communities. Sci Rep. 2022;12:1–12. 35982107 10.1038/s41598-022-18392-5 PMC9387884

[4] Wang QQ Xu R Volkow ND. Increased risk of COVID-19 infection and mortality in people with mental disorders: analysis from electronic health records in the United States. World Psychiatry. 2021;20:124–30. 33026219 10.1002/wps.20806 PMC7675495

[5] Figueroa JF Wadhera RK Lee D. Community-level factors associated with racial and ethnic disparities in covid-19 rates in Massachusetts. Health Aff (Millwood). 2020;39:1984–92. 32853056 10.1377/hlthaff.2020.01040 PMC8928571

[6] Johnson KS. Racial and ethnic disparities in palliative care. J Palliat Med. 2013;16:1329. 24073685 10.1089/jpm.2013.9468 PMC3822363

[7] Wachterman MW Sommers BD. Dying poor in the US – disparities in end-of-life Care. JAMA. 2021;325:423–4. 33528526 10.1001/jama.2020.26162

[8] Briedé S Van Goor HMR De Hond TAP. Code status documentation at admission in COVID-19 patients: a descriptive cohort study. BMJ Open. 2021;11:e050268. 10.1136/bmjopen-2021-050268 PMC8587534 34758991

[9] Mesfin N Fischman A Garcia MA. Predictors to forgo resuscitative effort during Covid-19 critical illness at the height of the pandemic: a retrospective cohort study. Palliat Med. 2021;35:1519–24. 34479453 10.1177/02692163211022622

[10] Katamreddy A Ye AM Vorchheimer DA. Exploring the changes in code status during the COVID-19 pandemic and the implications for future pandemic care. Am J Hosp Palliat Care. 2022;39:104990912210926. 10.1177/10499091221092699 PMC9038937 35452316

[11] Barnato AE Johnson GR Birkmeyer JD. Advance care planning and treatment intensity before death among black, hispanic, and white patients hospitalized with COVID-19. J Gen Intern Med. 2022;37:1–7. 10.1007/s11606-022-07530-4 PMC9002036 35412179

[12] Epler K Lenhan B O’Callaghan T. If your heart were to stop: characterization and comparison of code status orders in adult patients admitted with COVID-19. J Palliat Med. 2022;25:888–96. 34967678 10.1089/jpm.2021.0486 PMC9145568

[13] Moin EE Okin D Jesudasen SJ. Code status orders in patients admitted to the intensive care unit with COVID-19: a retrospective cohort study. Resusc Plus. 2022;10:100219. 35284847 10.1016/j.resplu.2022.100219 PMC8898738

[14] Neighborhood Atlas. 2015. https://www.neighborhoodatlas.medicine.wisc.edu [access date September 15, 2020].

[15] Kind AJH Jencks S Brock J. Neighborhood socioeconomic disadvantage and 30-day rehospitalization: a retrospective cohort study. Ann Intern Med. 2014;161:765–74. 25437404 10.7326/M13-2946 PMC4251560

[16] Kind AJH Buckingham WR. Making neighborhood-disadvantage metrics accessible – the neighborhood atlas. N Engl J Med. 2018;378:2456–8. 29949490 10.1056/NEJMp1802313 PMC6051533

[17] Dashti H Roche EC Bates DW. SARS2 simplified scores to estimate risk of hospitalization and death among patients with COVID-19. Sci Rep. 2021;11:4945. 33654180 10.1038/s41598-021-84603-0 PMC7925678

[18] Wynants L Calster BV Collins GS. Prediction models for diagnosis and prognosis of covid-19: systematic review and critical appraisal. BMJ. 2020;369:26. 10.1136/bmj.m1328 PMC7222643 32265220

[19] Jones AE Trzeciak S Kline JA. The Sequential Organ Failure Assessment score for predicting outcome in patients with severe sepsis and evidence of hypoperfusion at the time of emergency department presentation. Crit Care Med. 2009;37:1649–54. 19325482 10.1097/CCM.0b013e31819def97 PMC2703722

[20] Charlson ME Pompei P Ales KL. A new method of classifying prognostic comorbidity in longitudinal studies: development and validation. J Chronic Dis. 1987;40:373–83. 3558716 10.1016/0021-9681(87)90171-8

[21] Acosta AM Garg S Pham H. Racial and ethnic disparities in rates of COVID-19–associated hospitalization, intensive care unit admission, and in-hospital death in the United States from March 2020 to February 2021. JAMA Netw Open. 2021;4:e2130479. 34673962 10.1001/jamanetworkopen.2021.30479 PMC8531997

[22] Burgio KL Williams BR Dionne-Odom JN. Racial differences in processes of care at end of life in VA medical centers: planned secondary analysis of data from the BEACON trial. J Palliat Med. 2016;19:157–63. 26840851 10.1089/jpm.2015.0311 PMC4939451

[23] Perry LM Walsh LE Horswell R. Racial disparities in end-of-life care between black and white adults with metastatic cancer. J Pain Symptom Manage. 2021;61:342–349.e1. 32947018 10.1016/j.jpainsymman.2020.09.017 PMC8100959

[24] Brown CE Engelberg RA Sharma R. Race/ethnicity, socioeconomic status, and healthcare intensity at the end of life. J Palliat Med. 2018;21:1308–16. 29893618 10.1089/jpm.2018.0011 PMC6154447

[25] Kutney-Lee A Smith D Thorpe J. Race/ethnicity and end-of-life care among veterans. Med Care. 2017;55:342–51. 27579913 10.1097/MLR.0000000000000637

[26] Nelson A. Unequal treatment: confronting racial and ethnic disparities in health care. J Natl Med Assoc. 2002;94:666–8. 12152921 PMC2594273

[27] Johnson KS Kuchibhatla M Tulsky JA. What explains racial differences in the use of advance directives and attitudes toward hospice care? J Am Geriatr Soc. 2008;56:1953–8. 18771455 10.1111/j.1532-5415.2008.01919.x PMC2631440

[28] McCarty TR Hathorn KE Redd WD. How do presenting symptoms and outcomes differ by race/ethnicity among hospitalized patients with COVID-19 infection? Experience in Massachusetts. Clin Infect Dis. 2021;73:e4131–8. 32827436 10.1093/cid/ciaa1245 PMC7499493

[29] Wang Z Zheutlin A Kao YH. Hospitalised COVID-19 patients of the Mount Sinai Health System: a retrospective observational study using the electronic medical records. BMJ Open. 2020;10:e040441. 10.1136/bmjopen-2020-040441 PMC7592304 33109676

[30] Gu T Mack JA Salvatore M. Characteristics associated with racial/ethnic disparities in COVID-19 outcomes in an academic health care system. JAMA Netw Open. 2020;3:e2025197. 33084902 10.1001/jamanetworkopen.2020.25197 PMC7578774

[31] Cates J Lucero-Obusan C Dahl RM. Risk for in-hospital complications associated with COVID-19 and influenza – veterans health administration, United States, October 1, 2018–May 31, 2020. MMWR Morb Mortal Wkly Rep. 2020;69:1528–34. 33090987 10.15585/mmwr.mm6942e3 PMC7583498

[32] Nayar P Qiu F Watanabe-Galloway S. Disparities in end of life care for elderly lung cancer patients. J Community Health. 2014;39:1012–9. 24643730 10.1007/s10900-014-9850-x

[33] Hardy D Chan W Liu CC. Racial disparities in the use of hospice services according to geographic residence and socioeconomic status in an elderly cohort with nonsmall cell lung cancer. Cancer. 2011;117:1506–15. 21425152 10.1002/cncr.25669

[34] Binder AF Huang GC Buss MK. Uninformed consent: do medicine residents lack the proper framework for code status discussions? J Hosp Med. 2016;11:111–6. 26471452 10.1002/jhm.2497

[35] Calam B Far S Andrew R. Discussions of “code status” on a family practice teaching ward: what barriers do family physicians face? CMAJ. 2000;163:1255–59. 11107460 PMC80314

[36] Patel NK Passalacqua SA Meyer KN. Full code to do-not-resuscitate: culturally adapted palliative care consultations and code status change among seriously ill hispanic patients. Am J Hosp Palliat Care. 2022;39:791–7. 34467766 10.1177/10499091211042305 PMC12776030

[37] Pan C Palathra B Pelissier L. The ethnic divide: what is the association between inpatient palliative care consultations and code status among whites, Asians, Hispanics and Blacks? (S536). J Pain Symptom Manage. 2022;63:925.

[38] Patneaude A Kett J. Cultural responsiveness and palliative care during the COVID-19 pandemic. Palliat Med Rep. 2020;1:171. 34223473 10.1089/pmr.2020.0049 PMC8241343

[39] Valdez AC Iftekhar EN Oliu-Barton M. Europe must come together to confront omicron. BMJ. 2022;376:o90. 35027352 10.1136/bmj.o90

[40] Priesemann V Balling R Bauer S. Towards a European strategy to address the COVID-19 pandemic. Lancet. 2021;398:838–9. 34384539 10.1016/S0140-6736(21)01808-0 PMC8352491

[41] Czypionka T Iftekhar EN Prainsack B. The benefits, costs and feasibility of a low incidence COVID-19 strategy. Lancet Reg Health Eur. 2022;13:100294. 35005678 10.1016/j.lanepe.2021.100294 PMC8720492

