# Supplementary material for: Evaluating disparities in code status designation among patients admitted with COVID-19 at a quaternary care center early in the pandemic
Source: Medicine (Baltimore). 2023 Jul 28;102(30):e34447. doi: 10.1097/MD.0000000000034447 (PMC10376097; doi:10.1097/MD.0000000000034447)
Supplement: Supplementary file 1 [file medi-102-e34447-s001.pdf]

Supplemental Table 1: Univariable analysis of criteria associated with a final code status other than full code (DNR/DNI or Comfort Measures Only). Using an initial cut-off of p=0.10, variables used at the start of building the multivariable analysis are highlighted in blue. CI: Confidence Interval; CRP: C-reactive protein; BUN: Blood urea nitrogen; ALC: Absolute lymphocyte count; CK: Creatinine kinase; SOFA: Sequential Organ Failure Assessment; CCI: Charlson Comorbidity Index; ICU: Intensive Care Unit; HIV: Human immunodeficiency virus; ADI: Area Deprivation Index

	**Odds Ratio (95% CI)**	**p value**
**Age, yrs (per year)**	1.1 (1.1-1.1)	<0.001
**Female Sex (reference: male sex)**	1.5 (0.8-1.4)	0.71
**CRP, mg/L (per unit)**	1.0 (1.0-1.0)	0.73
**BUN, mg/dL (per unit)**	1.1 (1.0-1.6)	<0.001
**ALC, K/uL (per unit)**	0.8 (0.6-1.0)	0.07
**CK, U/L (per unit)**	1.0 (1.0-1.0)	0.14
**D-dimer, ng/mL (per unit)**	1.0 (1.0-1.0)	<0.001
**Procalcitonin, ng/mL (per unit)**	1.1 (1.0-1.1)	0.02
**Troponin, ng/L (per unit)**	1.0 (1.0-1.0)	<0.001
**Ferritin, ug/L (per unit)**	1.0 (1.0-1.0)	0.09
**SOFA (per unit in the score)**	1.2 (1.1-1.3)	<0.001
**CCI (per unit in the score)**	1.9 (1.8-2.1)	<0.001
**Hypotension (SBP <90mmHg) on admission (reference: no hypotension on admission)**	3.9 (2.2-6.9)	<0.001
**Oxygen requirement on admission (reference: no oxygen requirement on admission)**	2.6 (2.9-3.4)	<0.001
**ICU stay (reference: no ICU stay)**	1.6 (1.2-2.1)	0.001
**Intubated (reference: not intubated)**	1.7 (1.3-2.2)	<0.001
**History of renal disease (reference: no history)**	3.7 (2.7-5.2)	<0.001
**History of lung disease (reference: no history)**	2.4 (1.9-3.2)	<0.001
**History of hypertension (reference: no history)**	3.6 (2.7-4.8)	<0.001
**History of diabetes (reference: no history)**	1.4 (1.1-1.8)	0.02
**History of congestive heart failure (reference: no history)**	6.6 (4.6-9.7)	<0.001
**History of stroke (reference: no history)**	5.3 (3.2-8.7)	<0.001
**History of malignancy (reference: no history)**	3.1 (2.2-4.4)	<0.001
**History of HIV (reference: no history)**	0.9 (0.3-2.9)	0.87
**BMI ≥30mg/kg^2 (reference: BMI <30mg/kg^2)**	0.7 (0.6-0.9)	0.01
**White race (reference: non-white)**	5.0 (3.8-6.7)	<0.001
**ADI (per unit in the score)**	0.9 (0.9-1.0)	0.03
**English primary language (reference: non-English)**	2.9 (2.1-4.1)	<0.001

**Homelessness (reference: domiciled)**	0.2 (0.1-0.8)	0.02
**Illicit drug use (opiates, cocaine, methamphetamines) (reference: no illicit drug use)**	0.5 (0.2-1.0)	0.05
**Current cigarette smoker (reference: no active cigarette use)**	0.9 (0.5-1.5)	0.62

Supplemental Table 2: Univariable analysis of criteria associated with a final code status of ”Comfort Measures Only”. Using an initial cut-off of p=0.10, variables used at the start of building the multivariable analysis are highlighted in blue. CI: Confidence Interval; CRP: C-reactive protein; BUN: Blood urea nitrogen; ALC: Absolute lymphocyte count; CK: Creatinine kinase; SOFA: Sequential Organ Failure Assessment; CCI: Charlson Comorbidity Index; ICU: Intensive Care Unit; HIV: Human immunodeficiency virus; ADI: Area Deprivation Index

	**Odds Ratio (95% CI)**	**p value**
**Age, yrs (per year)**	1.1 (1.1-1.1)	<0.001
**Female Sex (reference: male sex)**	0.7 (0.5-1.1)	0.10
**CRP, mg/L (per unit)**	1.0 (1.0-1.0)	0.002
**BUN, mg/dL (per unit)**	1.0 (1.0-1.1)	<0.001
**ALC, K/uL (per unit)**	0.8 (0.6-1.2)	0.22
**CK, U/L (per unit)**	1.0 (1.0-1.0)	0.05
**D-dimer, ng/mL (per unit)**	1.0 (1.0-1.0)	<0.001
**Procalcitonin, ng/mL (per unit)**	1.9 (1.0-1.2)	0.003
**Troponin, ng/L (per unit)**	1.0 (1.0-1.0)	<0.001
**Ferritin, ug/L (per unit)**	1.0 (1.0-1.0)	0.02
**SOFA (per unit in the score)**	1.3 (1.2-1.4)	<0.001
**CCI (per unit in the score)**	1.6 (1.5-1.7)	<0.001
**Hypotension (SBP <90mmHg) on admission (reference: no hypotension on admission)**	2.7 (1.4-5.1)	0.004
**Oxygen requirement on admission (reference: no oxygen requirement on admission)**	3.7 (2.5-5.4)	<0.001
**ICU stay (reference: no ICU stay)**	2.7 (1.9-3.9)	<0.001
**Intubated (reference: not intubated)**	2.4 (1.7-3.5)	<0.001
**History of renal disease (reference: no history)**	4.1 (2.8-6.0)	<0.001
**History of lung disease (reference: no history)**	2.1 (1.5-3.1)	<0.001
**History of hypertension (reference: no history)**	3.5 (2.3-5.3)	<0.001
**History of diabetes (reference: no history)**	1.8 (1.3-2.6)	0.001
**History of congestive heart failure (reference: no history)**	4.8 (3.2-7.2)	<0.001
**History of stroke (reference: no history)**	2.8 (1.6-4.9)	<0.001
**History of malignancy (reference: no history)**	2.2 (1.4-3.3)	<0.001
**History of HIV (reference: no history)**	0.5 (0.1-3.7)	0.48
**BMI ≥30mg/kg^2 (reference: BMI <30mg/kg^2)**	0.9 (0.7-1.4)	0.74
**White race (reference: non-white)**	3.7 (2.5-5.5)	<0.001
**ADI (per unit in the score)**	1.0 (0.9-1.1)	0.79
**English primary language (reference: non-English)**	2.2 (1.4-3.5)	0.001
**Homelessness (reference: domiciled)**	0.2 (0.03-1.5)	0.12

**Illicit drug use (opiates, cocaine, methamphetamines) (reference: no illicit drug use)**	0.7 (0.2-1.9)	0.42
**Current cigarette smoker (reference: no active cigarette use)**	0.8 (0.4-1.7)	0.51
